# Pluvial Drainage Patterns and Holocene Desiccation Influenced the Genetic Architecture of Relict Dace, *Relictus solitarius* (Teleostei: Cyprinidae)

**DOI:** 10.1371/journal.pone.0138433

**Published:** 2015-09-22

**Authors:** Derek D. Houston, R. Paul Evans, Dennis K. Shiozawa

**Affiliations:** 1 Department of Biology, Brigham Young University, Provo, Utah, United States of America; 2 Department of Microbiology and Molecular Biology, Brigham Young University, Provo, Utah, United States of America; University of Arkansas, UNITED STATES

## Abstract

Changing drainage patterns have played a significant role in the evolution of western North American aquatic taxa. Relict dace, *Relictus solitarius*, is a Great Basin endemic cyprinid with a native range that is restricted to four valleys in eastern Nevada. *Relictus solitarius* now occupies spring systems that are the remnants of Pleistocene-era pluvial lakes, although it may have occurred in the area for much longer. Here we use mitochondrial DNA sequence data to assess range-wide genetic diversity of *R*. *solitarius*, and to estimate divergence times to determine whether pluvial drainages played an important role in shaping intraspecific genetic diversity. Genetic diversification within *R*. *solitarius* began during the early to mid-Pleistocene, separating populations within two sets of valleys (Butte/Ruby and Goshute/Steptoe). Additional diversification in each of the two sets of valleys occurred more recently, in the mid- to late-Pleistocene. Holocene desiccation has further isolated populations, and each population sampled contains unique mtDNA haplotypes. Pluvial drainage patterns did contribute to the genetic structure observed within *R*. *solitarius*, but most of the intraspecific diversification does not appear to be associated with the Last Glacial Maximum. Holocene desiccation has also contributed to the observed genetic structure. The relict dace populations we sampled are all unique, and we recommend that future management efforts should strive to preserve as much of the genetic diversity as possible.

## Introduction

Western North American drainage patterns have been dramatically altered throughout the Cenozoic as a result of a myriad of geological processes and global climate shifts. The modern Great Basin comprises a series of endorheic hydrological basins (closed drainages with no outlet to the ocean). These basins formed as a result of the uplift and episodic extension of western North America, beginning in the Eocene. The westward extension of the Great Basin continued through the Miocene, and is still underway [1,2]. These drastic changes to the landscape sometimes disrupted regional hydrology. Shifting drainage patterns have affected the evolution of many western North American aquatic taxa through the resulting isolation of populations, or novel contact between those that were previously isolated [3–9]. Several western North American freshwater fishes exhibit deep genetic divergences associated with these ancient changes to the landscape [10–14], and many exhibit intra-specific cryptic genetic diversity [15–22].

Climatic oscillations during the Pleistocene also played a significant role in the evolution of the western North American biota by forcing organisms through a series of range contractions, expansions, and shifts as glacial ice sheets expanded and retracted [23]. The increased precipitation and decreased evaporation associated with periods of lower global temperatures during the Pleistocene resulted in the formation of pluvial lakes in many valleys within the Great Basin [24–26]. Pluvial lakes expanded during glacial periods and contracted during interglacial periods, often leaving prominent beach terraces above many Great Basin valley floors [1,27–29]. In some cases, rising lake levels resulted in aquatic connections between sub-basins within the Great Basin, which may have allowed the transfer of aquatic taxa [30,31]. Some aquatic taxa dispersed between sub-basins via such aquatic connections [18,32], while others do not appear to have done so [19,33,34]. Thus, the influence of pluvial lakes on genetic diversity of aquatic taxa may have been dependent on the ecological requirements and life histories of individual taxa. Additional phylogeographic studies of western North American freshwater fishes should yield further insight into how geological and climatic factors have influenced the evolution of aquatic taxa in the region.

**Fig 1:**
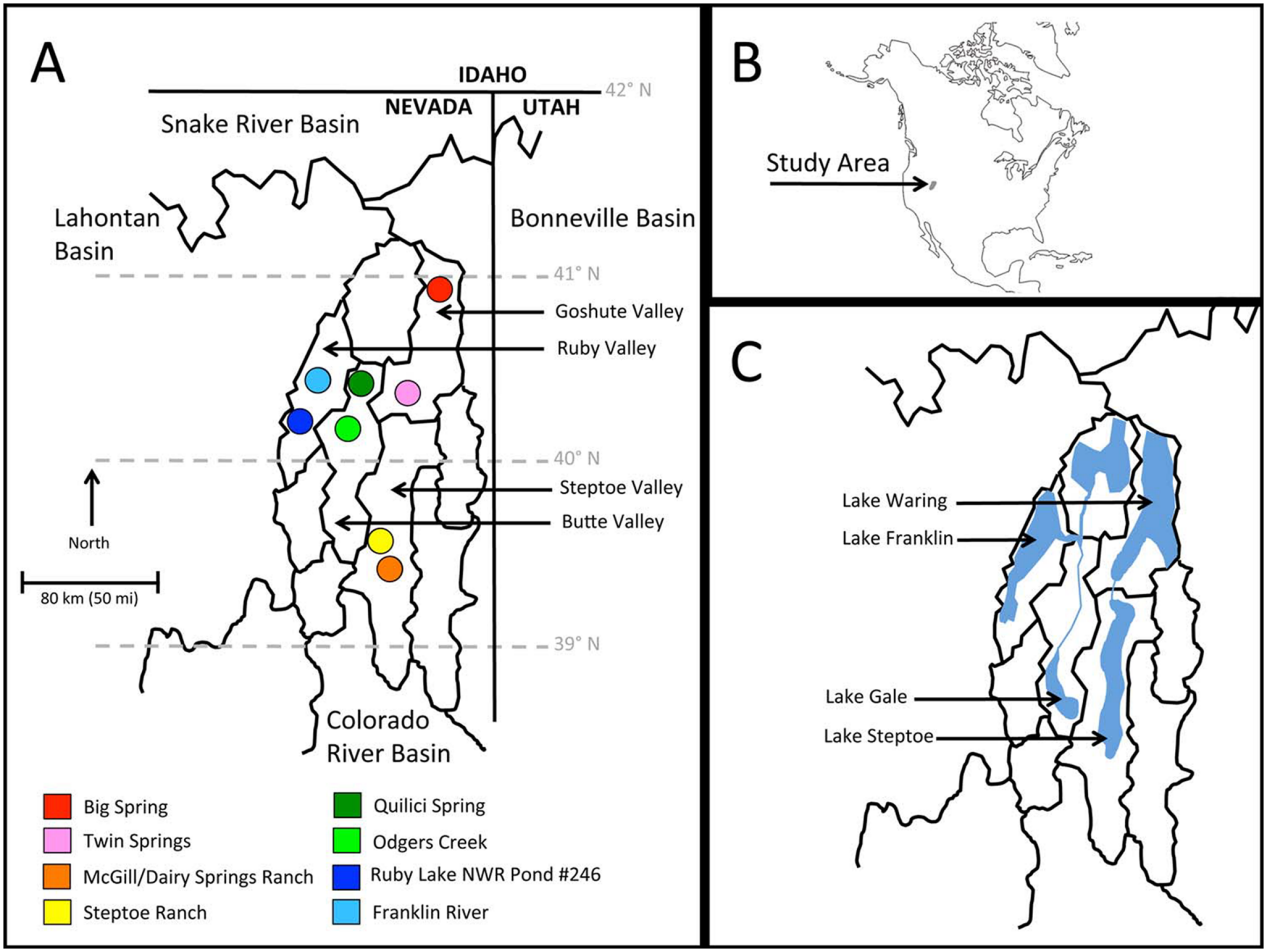
Distribution map for *R*. *solitarius*. A) Map showing the locations of eight relict dace populations that were included in this study. The natural species range of the relict dace is contained within the four valleys that are labeled. A colored circle represents each sampling locality, and the color of each circle varies so that subsequent figures can be interpreted more easily. Colors for each population are as follows: Big Spring (red), Twin Springs (pink), Franklin River (light blue), Ruby Lake National Wildlife Refuge Pond #246 (dark blue), Quilici Spring (dark green), Odgers Creek (light green), Steptoe Ranch (yellow), and McGill/Dairy Springs (orange). B) The location of the study area in western North America. C) Pluvial drainage systems that existed in the study area during the Pleistocene Epoch. Relict dace currently reside in spring systems that are considered to be remnants of these pluvial drainages.

The relict dace, *Relictus solitarius*, is a narrowly distributed Great Basin endemic cyprinid [35], and is the sole member of its genus. The entire native range of *R*. *solitarius* is confined to four closed basins in the north-central Great Basin: Butte Valley, Goshute Valley, Ruby Valley, and Steptoe Valley in eastern Nevada (Fig 1a and 1b). *Relictus solitarius* is the only native fish species that occurs in these valleys, and it occupies spring systems that are remnants of the pluvial lakes that formed in all four valleys during the Pleistocene [36]. Pluvial Lake Gale formed in Butte Valley, Lake Waring in Goshute Valley, Lake Franklin in Ruby Valley, and Lake Steptoe in Steptoe Valley (Fig 1c). *Relictus solitarius* is postulated to have evolved pre-Pliocene [36], a time frame that falls within confidence intervals surrounding divergence time estimates among these genera [12]. Hence, *R*. *solitarius* represents a species that may have arisen as a result of ancient geological changes to the landscape but has also been influenced by Pleistocene climate oscillations.

Geomorphological evidence shows inter-basin spillover connections between Butte Valley and Ruby Valley [37,38], thus indicating that Lake Gale and Lake Franklin were connected as part of the same pluvial drainage system during some lake high stands (Fig 1). Similarly, Steptoe Valley and Goshute Valley were connected as part of a separate pluvial drainage system via outflows northward from Lake Steptoe to Lake Waring (see Fig 1). Hubbs et al. [36] stated that an aquatic transfer allowing the dispersal of *R*. *solitarius* between pluvial Lake Franklin (Ruby Valley) and Lake Waring (Goshute Valley) likely occurred, although they did not speculate on the direction of this postulated dispersal event. No clear overflow connections exist between the two pairs of valleys (Butte/Ruby vs. Goshute/Steptoe), as satellite imagery does not show any signs of scarring similar to the aforementioned connections between the other valleys. However, other biogeographic signals demonstrate western North American aquatic connections for which there is little to no geological evidence [7,10,12,34].

The postulated connection between Lake Franklin (Ruby Valley) and Lake Waring (Goshute Valley) would have provided a Pleistocene-aged aquatic connection between the four valleys where *R*. *solitarius* currently resides. Hubbs et al. [36] hypothesized that *R*. *solitarius* was in just one valley (but did not speculate about which one) and dispersed among pluvial drainages through these aquatic connections in a short period of time. It was long thought that *R*. *solitarius* populations within these four valleys had not differentiated based on lack of morphological differences across the species’ range [7,36]. However, recent mtDNA evidence shows that some genetic differentiation has occurred [39], although the timing of diversification among *R*. *solitarius* populations has yet to be determined.

In this study, we used mtDNA sequence data to assess the range-wide genetic structure of *R*. *solitarius* and determine the timing of Pleistocene and Holocene impacts on that structure. Any inter-basin gene flow that occurred during the Wisconsin glaciation would have ceased as pluvial drainages dried with the onset of Holocene desiccation. If so, divergence times may correspond to a late-Pleistocene/early-Holocene time frame. Alternatively, it is plausible that diversification within *R*. *solitarius* occurred earlier in the Pleistocene, as pluvial lake levels fluctuated with glacial and inter-glacial cycles. Another alternative is that *R*. *solitarius* divergence times pre-date the Pleistocene, corresponding to ancient changes to the landscape and they did not disperse through pluvial drainage connections (or did disperse, resulting in secondary contact between clades). The latter alternative is plausible given divergence times between *Relictus* and other genera that are Pliocene in age with confidence intervals spanning from late-Miocene to mid-Pleistocene [12], and that there is no clear geomorphological evidence for connections between the pairs of valleys.

To assess the evolutionary relationships among populations of *R*. *solitarius*, we generated phylogenies using Bayesian inference and maximum likelihood. We also constructed a haplotype network to better visualize the finer-scale phylogeographic structure. We estimated divergence times among major clades within *R*. *solitarius* to determine whether splits were Pleistocene in age and, if so, whether they were in the early, middle or late-Pleistocene. We also explored genetic variation at hierarchical geographic levels to determine which level best explains the observed variation.

## Materials and Methods

### Sampling, DNA Extraction, and Polymerase Chain Reaction

We examined DNA from seventy-nine relict dace specimens from eight natural populations; two from each of the four valleys where relict dace naturally occur (S1 Table). The two Ruby Valley populations included in this study were chosen because they do not exhibit genetic admixture with speckled dace that have been introduced into some Ruby Valley springs [39]. Nevada Department of Wildlife (NDOW) officials and Otis Bay Ecological Consultant (OBEC) biologists collected samples under authorized permits and procedures. We used previously published cyt *b* sequences (GenBank #s are listed in S1 Table), and sequenced an additional mitochondrial gene, the NADH subunit 2 (ND2), for each relict dace individual. We amplified ND2 using primers ASN and ILE [40]. Protocols for PCR amplifications are described in detail by Houston et al. [39].

### Sequencing, Alignment and Analysis of Molecular Variation

We used purified PCR products as templates for cycle-sequencing reactions, and carried out Sanger sequencing according to methods described by Houston et al. [39], the only difference being the primers used (ASN and ILE). For the previously published cyt *b* sequences, we used aligned sequences from Houston et al. [39]. We aligned the newly generated ND2 sequences using the automatic assembly feature in Sequencher v.4.8 (Gene Codes Corporation), then visually inspected the aligned sequences and made corrections manually, using a speckled dace (*Rhinichthys osculus*) ND2 sequence (GenBank: EU158191) and amino acid sequence as references. We concatenated both mtDNA genes prior to performing phylogenetic analyses after a likelihood ratio test showed no significant difference between models selected for each gene separately (p > 0.05). Sequences from northern pikeminnow (*Ptychocheilus oregonensis*) and hardhead (*Mylopharodon conocephalus*) were included in the alignment as outgroup taxa because they are closely related to relict dace [12,13,41], and genomic DNA samples were readily available. There were no gaps in the final alignment.

We performed analysis of molecular variance (AMOVA) using Arlequin v.3.11 [42]. Samples were grouped hierarchically by region (i.e., pluvial drainage system: Lake Gale/Lake Franklin versus Lake Steptoe/Lake Waring), and by populations within regions (i.e., sampling localities).

### Phylogenetic Analyses

We used jModeltest2 v.2.1.4 [43] as implemented in PHyML v.3.0 [44] to determine an appropriate model of sequence evolution for the concatenated mtDNA data. We estimated phylogenies using maximum likelihood (ML). We performed ML analysis using Treefinder version of 2008 [45] including 1000 bootstrap replicates to estimate nodal support.

We performed Bayesian analysis using MrBayes v.3.2 [46] employing a Markov Chain Monte Carlo approach with two independent runs, each with one cold chain and three heated chains. We ran Bayesian analysis for 10,000,000 generations, sampling every 1000 generations, and evaluated whether the analysis achieved stationarity and proper mixing using Tracer v.1.6 [47]. To get proper mixing of chains, we lowered the temperature setting from the default value T = 0.20 to T = 0.05. We obtained posterior probabilities using the majority rule consensus for the remaining topologies after discarding the first 2,500,000 generations as burn-in.

To better visualize intraspecific mtDNA genetic diversity, we generated a haplotype network using TCS v.1.2.1 [48], using the default connection limit of 0.95.

### Molecular Dating Estimation

We generated molecular dating estimates using the uncorrelated lognormal relaxed clock in the program BEAST 1.7.5 [49]. We used the coalescent constant population size model to set the prior on the tree. For the mutation rate prior distribution, we used a lognormal distribution with a mean mutation rate of 1.4% sequence divergence per million years, allowing for a range of 1.0% to 2.0% sequence divergence per million years, a range that is consistent with mutation rates in these mtDNA genes for other small-bodied cyprinids [10,13,17,50]. We ran the MCMC chain for 50,000,000 generations, sampling every 1000 generations, and discarded the first 5,000,000 as burn-in. We verified that the program reached stationarity, had proper mixing of chains, and reached appropriate ESS values using Tracer v.1.6 [47] and annotated trees using TreeAnnotator v1.7.2 [49].

## Results

### Sequencing, Alignment, and Analysis of Molecular Variation

DNA sequencing yielded 1047 base pairs of ND2 that we combined with 1140 base pairs of cyt *b* (previously published) from 79 relict dace individuals representing 8 populations. There were 2187 total characters in the final alignment, 458 of which were variable. All DNA sequences have been made publicly available in GenBank (accession numbers are listed in S1 Table). Forty-six unique mtDNA haplotypes were found among all relict dace we sampled.

The results of AMOVA (Table 1) show that the highest proportion of genetic variation (81.8%) is explained by variation within relict dace populations, with the second highest proportion explained by genetic structuring among sampling localities (17.4%), and a minimal amount (0.76%) explained by geographical structure between groups (i.e., pluvial drainage systems).

**Table 1:** AMOVA Results. Populations were categorized as either Ruby/Butte or Goshute/Steptoe in accordance with phylogenetic placement in one of two clades. Within population variation explains the majority of the molecular variation observed within these samples of relict dace.

Source of Variation	d.f.	Sum of squares	Variance components	% of molecular variation explained
Among groups (i.e., pluvial drainages)	1	1.419	0.00378	0.76
Among populations within groups (i.e., sampling localities)	6	7.614	0.08709	17.41
Within populations	71	29.056	0.40923	81.83
Total	78	38.089	0.50010	100

### Phylogenetic Analyses and Molecular Dating

**Fig 2:**
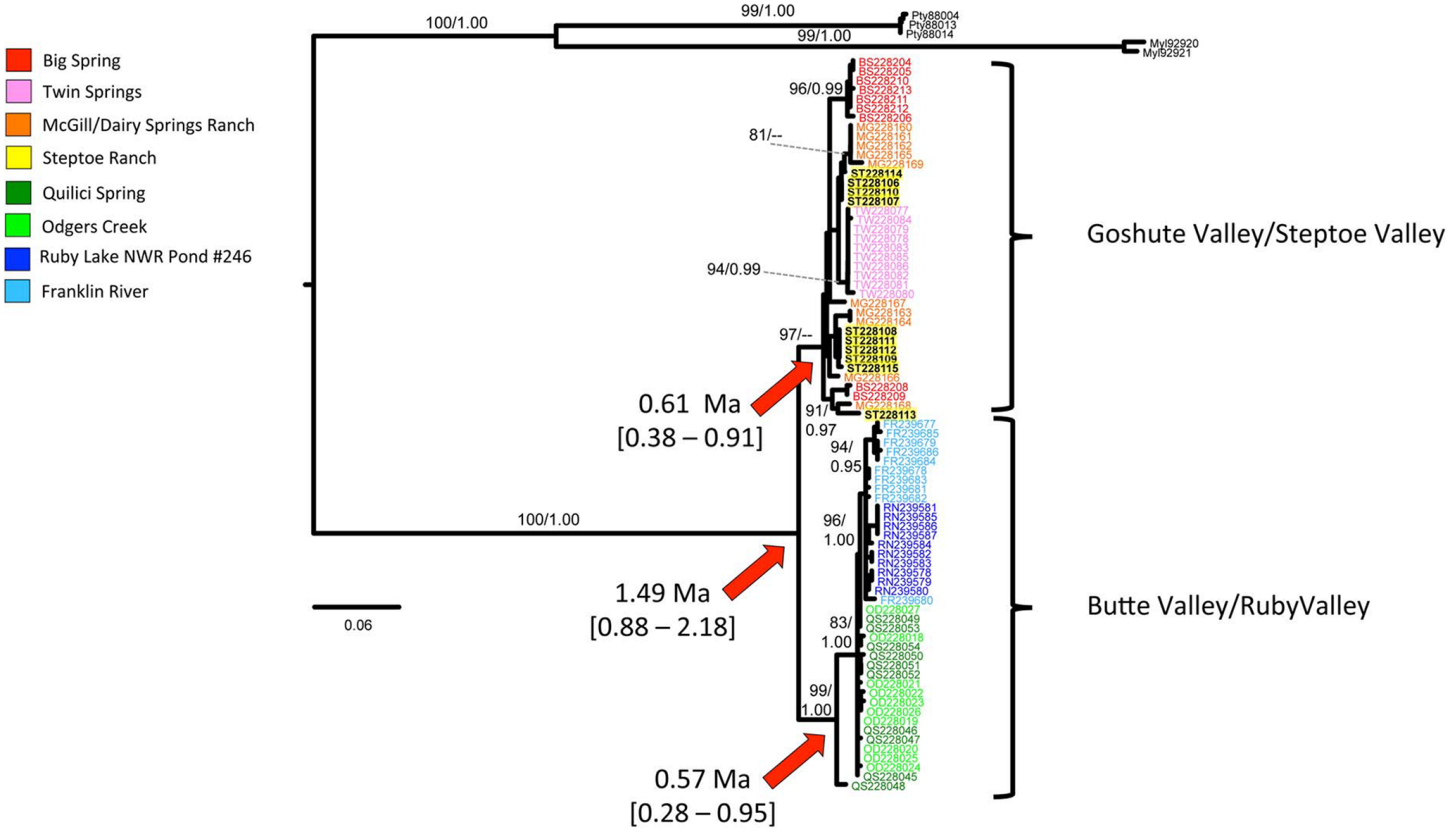
Relict dace phylogeny. Phylogeny based on concatenated mtDNA sequences. Maximum likelihood bootstraps and Bayesian posterior probabilities are listed above each node that they support (in that order). Divergence time estimates are marked with red arrows, and 95% credible intervals for each estimate are listed in brackets below the mean value.

The GTR+I model of sequence evolution was selected by jModeltest as the best fit for the concatenated mtDNA dataset under both the Akaike Information Criterion and the Bayesian Information Criterion. The phylogenies produced by ML and Bayesian analyses were similar, so only the ML phylogeny is shown (Fig 2), but both ML bootstrap values and Bayesian posterior probabilities are mapped above branches to illustrate nodal support. Relict dace from populations in Butte Valley and Ruby Valley form a well-supported monophyletic clade (Fig 2). Individuals from Goshute Valley and Steptoe Valley form a clade that was supported by ML analysis, but not Bayesian inference (Fig 2). However, the Goshute/Steptoe clade was recovered in our other analyses, including the haplotype network, and our Bayesian molecular dating estimates (see below). Collapsing that node would lead to a series of lineages stemming from a basal polytomy for the species.

Molecular dating estimates show that diversification within *R*. *solitarius* began approximately 1.5 million years ago, in the mid-Pleistocene (Fig 2). Diversification within the Goshute/Steptoe and the Ruby/Butte clades began later in the Pleistocene, with divergence time estimates of 0.61 Ma and 0.57 Ma, respectively (Fig 2).

## Discussion

Pluvial drainage patterns shaped the early genetic diversification among populations of *R*. *solitarius*. Divergence time estimates show that the initial intraspecific divergence between the two main clades began in the early to mid-Pleistocene, with subsequent diversification within phylogenetic clades occurring later in the Pleistocene (Fig 2). It is well established that there is uncertainty with divergence time estimates based solely on mtDNA data, and the use of additional unlinked markers would enhance our understanding of divergence times among clades of *R*. *solitarius*. Nonetheless, estimates based on mtDNA can still be informative for general time frames, and the 95% credibility intervals surrounding these estimates place the divergence times for *R*. *solitarius* well within the Pleistocene. This supports the hypothesis that pluvial drainages connected populations and allowed for gene flow, although most of those connections do not appear to be associated with lake high stands during the Last Glacial Maximum (~18 Ka).

**Fig 3:**
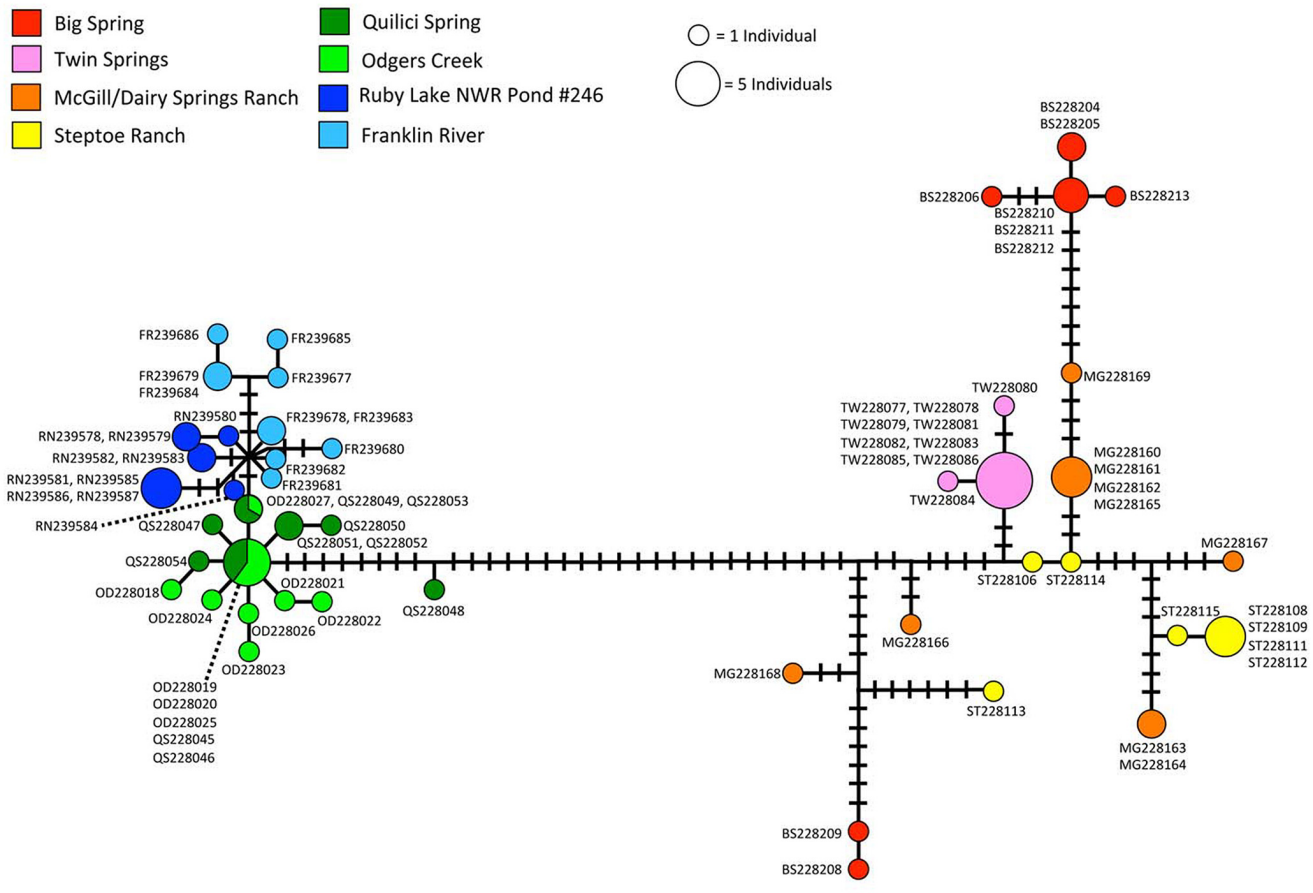
Relict dace mtDNA haplotype network. Haplotype network showing the intraspecific genetic diversity of relict dace populations throughout the species’ native range. Circles represent unique haplotypes, and the size of the circle represents the number of individuals that carry that haplotype. Hash marks between haplotypes represent base changes. Colors represent sampling localities.

Holocene desiccation and the resultant isolation of populations also appears to have greatly influenced genetic structure within *R*. *solitarius*. The results of AMOVA indicate that the majority of the genetic diversity within the species (~82%) is explained by within population variation (Table 1). The haplotype network shows that only the two populations in Butte Valley (Odgers Creek and Quilici Spring) share haplotypes, whereas all other populations contained unique haplotypes (Fig 3).

The haplotype network also shows a marked genetic distance between haplotypes carried by Butte/Ruby individuals and haplotypes carried by Goshute/Steptoe individuals (Fig 3). This is reflected in the deep separation between the two lineages in the phylogenetic analysis (Fig 2). The two lineages appear to have over a million years of isolation. This suggests that any connection between Ruby and Butte valleys on the west and Steptoe and Goshute valleys on the east would have been severed by the mid-Pleistocene.

The close genetic distances among Butte/Ruby haplotypes is consistent with a hypothesized Pleistocene aquatic connection [36] between pluvial Lake Waring (Goshute Valley) and pluvial Lake Franklin (Ruby Valley). That connection should have allowed the dispersal of *R*. *solitarius* between the two basins. Divergence time estimates show that the Ruby/Butte clade began diversifying in the mid- to late-Pleistocene (Fig 2). This implies that a connection between Butte and Ruby valleys could have been active during that time. The initial divergence between these two basins appears to be represented by a haplotype that occurs in Quilici Spring of Butte Valley (Fig 2), with a much more diverse and further diversification occurring in both Butte and Ruby valleys. The haplotype network (Fig 3) indicates that the Quilici Spring haplotype from the initial split connects to haplotypes in both Quilici Spring and Odgers Creek of Butte Valley and that this Butte Valley lineage then connects to haplotypes carried by the Ruby Valley relict dace, as would be expected if a founding haplotype from Butte Valley invaded Ruby Valley and later diversified. Hypotheses regarding dispersal between these areas can be better tested using additional unlinked markers and more sophisticated phylogeographic analyses.

Haplotypes in the Goshute/Steptoe individuals show a greater genetic diversity and distance between haplotypes in comparison to the Butte/Ruby individuals (Fig 3). The haplotypes also show a more complex pattern of relatedness on the phylogeny (Fig 2). This indicates that relict dace populations in Goshute and Steptoe Valleys have responded to Pleistocene events differently than Butte/Ruby populations. Haplotypes within these populations are genetically distinct, but populations are not monotypic. This pattern seems to reflect mid-Pleistocene exchange of haplotypes among populations in Goshute and Steptoe Valleys, followed by isolation, but without extensive lineage extinctions. This hypothesis would also be better tested using additional unlinked markers.

It is possible that genetic drift associated with population bottlenecks may have played a role in diversification of relict dace by increasing mutation rates [51]. If this was the case, our divergence time estimates may be older than the actual divergence times because of the accelerated rate. However, the allowed range surrounding our mutation rate prior should account for such a scenario. Moreover, such a scenario would still be consistent with diversification among *R*. *solitarius* populations occurring during the Pleistocene, but would push diversification among relict dace populations closer toward the late Pleistocene.

*Relictus solitarius* is not the only endemic Great Basin fish species that has experienced pronounced post-Pleistocene habitat fragmentation. Northern leatherside chub, *Lepidomeda copei*, once widely distributed, now occurs in isolated populations due to a combination of natural processes and anthropogenic disturbances [52]. Similarly, populations of Tui chub, *Siphateles bicolor*, appear to have been interconnected during the Pleistocene but now exhibit genetic structuring consistent with subsequent habitat fragmentation [53]. Habitat fragmentation of native fish species will likely continue as water loss is expected to increase in the Great Basin as the global climate changes [54]. Therefore, it is important to manage Great Basin fish populations accordingly. Relict dace populations across the species range exhibit pronounced genetic diversity, contrary to what was previously assumed based on morphology [7,36], but consistent with previous mtDNA results [39]. Populations in Goshute and Steptoe valleys exhibit the greatest amount of genetic diversity, but these two valleys are distinct from populations in Ruby and Butte valleys, which also contain many unique haplotypes. Typically at least one of three criteria should be met for the designation of Evolutionary Significant Units (ESUs): 1) current geographic isolation; 2) genetic differentiation in neutral markers; 3) local phenotypic adaptation [55,56]. Given that Holocene desiccation has effectively isolated these populations, coupled with numerous unique haplotypes within them, and high F_ST_ values among them [39], these relict dace populations clearly meet the first two criteria to be considered ESUs, but we currently do not have data to properly assess the third. Thus, these populations should be managed in a way that maximizes the preservation of the species-wide genetic diversity.

## Conclusions

Pluvial drainage patterns did contribute to diversification among relict dace populations that occurred during the Pleistocene, but early genetic divergences are not associated with the Last Glacial Maximum. Holocene desiccation has isolated populations and contributed to the overall genetic structure. All eight populations included in this study are genetically unique. These populations should be managed in a way that maximizes the preservation of the species-wide genetic diversity, particularly given the increasing demands and influences of anthropogenic activities on aquatic resources in western North America.

## Supporting Information

S1 Table Sampling localities. Location information for the eight populations of relict dace included in this study. Contemporary drainage is listed for each location, along with pluvial drainage and population names (abbreviations are listed in parentheses after the name of each sampling locality). BYU accession numbers, the number of individuals sampled from each populations, and GenBank accession numbers for both the cyt *b* and ND2 genes are also provided. Outgroup sampling localities are also listed.
(DOCX)
