# Supplementary material for: Pluvial Drainage Patterns and Holocene Desiccation Influenced the Genetic Architecture of Relict Dace, Relictus solitarius (Teleostei: Cyprinidae)
Source: PLoS One. 2015 Sep 22;10(9):e0138433. doi: 10.1371/journal.pone.0138433 (PMC4579093; doi:10.1371/journal.pone.0138433)
Supplement: S1 Table — Location information for the eight populations of relict dace included in this study. Contemporary drainage is listed for each location, along with pluvial drainage and population names (abbreviations are listed in parentheses after the name of each sampling locality). BYU accession numbers, the number of individuals sampled from each populations, and GenBank accession numbers for both the cyt b and ND2 genes are also provided. Outgroup sampling localities are also listed. (DOCX) [file pone.0138433.s001.docx]

| Drainage Basin | Sampling Locality | Pluvial Drainage | Latitude/  Longitude | BYU # | N | GenBank Accession # |
| --- | --- | --- | --- | --- | --- | --- |
| *Relictus solitarius* | |  |  |  |  |  |
| Butte Valley | Odgers Creek (OD) | Lake Gale | 40.224 N,  115.014 W | 228018 – 228027 | 10 | cyt *b*: JN997026 – JN997035  ND2: KR053471 – KR053480 |
|  | Quilici Spring (QS) | Lake Gale | 40.331 N,  115.060 W | 228045 – 228054 | 10 | cyt *b*: JN997036 – JN997045  ND2: KR053481 – KR053490 |
| Goshute Valley | Big Springs (BS) | Lake Waring | 40.967 N,  114.515 W | 228204 – 228206,  228208 – 228213 | 9 | cyt *b*: JN996870 – JN996878  ND2: KR053452 – KR053460 |
|  | Twin Springs (TW) | Lake Waring | 40.352 N,  114.829 W | 228077 – 228086 | 10 | cyt *b*: JN997073 – JN997082  ND2: KR053501 – KR053510 |
| Ruby Valley | Franklin River (FR) | Lake Franklin | 40.532 N,  115.211 W | 239677 – 239686 | 10 | cyt *b*: JN996889 – JN996898  ND2: KR053511 – KR053520 |
|  | Ruby Lake NWR Pond #246 (RN) | Lake Franklin | 40.077 N,  115.528 W | 239578 – 239587 | 10 | cyt *b*: JN996974 – JN996982, JN996993  ND2: KR053526 – KR053535 |
| Steptoe Valley | McGill/Dairy Springs Ranch (MG) | Lake Steptoe | 39.431 N,  114.808 W | 228160 – 228169 | 10 | cyt *b*: JN996924 – JN996933  ND2: KR053461 – KR053470 |
|  | Steptoe Ranch (ST) | Lake Steptoe | 39.534 N,  114.912 W | 228106 – 228115 | 10 | cyt *b*: JN997053 – JN997062  ND2: KR053491 – KR053500 |
| *Mylopharodon conocephalus* | |  |  |  |  |  |
| Sacramento River | Deer Creek, CA | N/A | 39.938 N, 122.052 W | 92920 – 92921 | 2 | cyt *b*: EU747217 – EU747218  ND2: KR053521 – KR053522 |
| *Ptychocheilus oregonensis* | |  |  |  |  |  |
| Columbia River | Row River, OR | N/A | 43.784 N,  122.954 W | 88004, 88013, 88014 | 3 | cyt *b*: EU747203, EU747207, EU747213  ND2: KR053523 – KR053525 |
